# The Economic Cost of Suicide and Non-Fatal Suicide Behavior in the Australian Workforce and the Potential Impact of a Workplace Suicide Prevention Strategy

**DOI:** 10.3390/ijerph14040347

**Published:** 2017-03-27

**Authors:** Irina Kinchin, Christopher M. Doran

**Affiliations:** 1 Centre for Indigenous Health Equity Research, School of Health, Medical and Applied Sciences, Psychology and Public Health Department, Cairns Campus of the CQUniversity, CQUniversity Australia, Cairns 4870, Australia; 2 Centre for Indigenous Health Equity Research, School of Health, Medical and Applied Sciences, Psychology and Public Health Department, Brisbane Campus of the CQUniversity, CQUniversity Australia, Brisbane 4000, Australia; c.doran@cqu.edu.au

**Keywords:** suicide, non-fatal suicide behavior, mental health, intentional self-harm, impact, cost, NCIS, workforce, prevention, economic

## Abstract

Suicide and non-fatal suicide behavior (NFSB) are significant problems faced by most countries. The objective of this research is to quantify the economic cost of suicide and NFSB in the Australian workforce and to examine the potential impact of introducing a workplace suicide prevention intervention to reduce this burden. The analysis used the best available suicide data, a well-established costing methodology, and a proven workplace intervention. In 2014, 903 workers died by suicide, 2303 workers harmed themselves resulting in full incapacity, and 11,242 workers harmed themselves resulting in a short absence from work. The present value of the economic cost of suicide and NFSB is estimated at $6.73 billion. Our analysis suggests the economic benefit of implementing a universal workplace strategy would considerably outweigh the cost of the strategy. For every one dollar invested, the benefits would be in excess of $1.50 ($1.11–$3.07), representing a positive economic investment. All variations of the key parameter hold the positive benefit-cost ratio. Rates of suicide and NFSB are far too high in Australia and elsewhere. More needs to be done to reduce this burden. Although workplace strategies are appropriate for those employed, these interventions must be used within a multifaceted approach that reflects the complex nature of self-harming behavior.

## 1. Introduction

Suicide and non-fatal suicide behavior (NFSB) are significant problems faced by most countries [1]. A suicide is a deliberate act of self-harm taken with the expectation that it will be fatal [2]. NFSB is defined as suicidal thoughts, plans, and attempts to die or inflict bodily harm [2]. NFSB is far more common than fatal suicide events and it is currently believed that for every death by suicide, there are between 10 and 20 attempted suicides [1]. The World Health Organization (WHO) reports that over 800,000 people die due to suicide every year, suggesting that there may be as many as 16 million people who attempt suicide [1].

In Australia, suicide is a major cause of death among people of working age [3]. In 2014, it was the leading cause of death in males aged 25–44 years and females aged 25–34 years [4]. Although evidence suggests that employment lowers the overall risk of suicide [5], it is estimated that between 20%–30% of the workforce will suffer from a serious mental health problem and for every employee who dies by suicide, another 10–20 will make a suicide attempt, with 17% resulting in a permanent disability and 83% no disability [1].

Suicide and NFSB have significant flow-on effects impacting the lives of any number of individuals—from family to friends, colleagues, clinicians, first responders, coronial staff, volunteers of bereavement support services, and other associates—who inevitably suffer intense and conflicted emotional distress in response to such behavior. Further, the economic impacts are significant and can include loss of productive capacity and earnings.

A few international studies have examined the economic loss and burden of suicide and suicidal behavior including studies in Canada, Ireland, New Zealand, and the United States [6,7,8,9,10,11,12]. Fewer studies are available in Australia [13,14,15], particularly among the Australian workforce [15]. While the emotional burden of suicide and suicidal behavior is difficult to estimate, the economic cost can help to raise awareness and inform the national call to preventive actions [7].

Unfortunately, the prevention of suicide has not been adequately addressed in society or the workforce, due to a lack of awareness of suicide as a major problem and the taboo in many societies to discuss it openly [1,16]. Mann et al. (2005) [17] conducted a systematic review of suicide prevention strategies and found that, overall, a range of National suicide prevention strategies have been proposed despite knowledge deficits about the effectiveness of some common key components. The authors suggest that the most promising interventions are physician education, means restriction (i.e., reducing access to lethal methods), and gatekeeper education (i.e., where the roles of gatekeepers are formalized and pathways to treatment are readily available). In a review undertaken by Doran et al. (2013) [18] for the New South Wales Mental Health Commission, it was noted that several experts point to the need for workplaces to become better equipped to handle psychological stress within their own companies. If employers were more aware of the economic consequences of the impact of mental disorders on their employees, the workplace could provide an ideal setting for mental health promotion and prevention. Hilton et al. (2008) [19] suggests that effective treatment for mental health problems yields substantial increases in employee productivity and would be a sound economic investment for employers.

In the recent research Doran et al. [20,21] estimated the economic cost and the impact of prevention of self-harm and suicide in the Australian construction industry using the costing methodology endorsed by the National Industry Commission [22] and applied over the years by the Australian National Occupational Health and Safety Commission [23]. The objective of this study is to draw on the past research [20,21,24] and validated costing methodology [15,22,23] to: (1) quantify the economic cost of suicide and non-fatal suicide behavior (NFSB) in the Australian workforce in one year; and (2) to model the potential impact to the society of introducing a workplace suicide prevention intervention that aims to reduce this harm.

## 2. Materials and Methods

### 2.1. Suicide Data

Suicide data were obtained from the National Coronial Information System (NCIS) for 2014. NCIS is a national internet based data storage and retrieval system for Australian coronial cases, established in 2001 [25]. NCIS is utilized by coroners, government agencies, and researchers for identifying cases for death investigation, research, and to monitor external causes of death in Australia.

### 2.2. Costing Methodology

The costing analysis relied on a methodology developed by the Industry Commission [22], refined by the Australian National Occupational Health and Safety Commission [23], and recently applied by Safe Work Australia [26,27]. The costing framework identifies direct and indirect costs for a range of economic agents (including employers, workers, and the government) segregated by severity (Table 1). In the current analysis, we use three levels of severity—short absence from work, full incapacity, and fatality. We assume that for every 15 suicide attempts there is one fatality, and from the 15 attempts, 3 (17%) are classified as full incapacity and 12 (83%) classified as short absence. This assumption is supported by research in Australia [28,29]. Corresponding duration of absence (for use in calculation of production disturbance costs) are 0.2 weeks for short absence; and 2.6 weeks for full incapacity and fatality [26].

Six cost groups were used to derive the total cost of suicide and NFSB: production disturbance costs; human capital costs; medical costs; administrative costs; other costs; and transfer costs (Table 2).

#### 2.2.1. Production Disturbance Costs

Production disturbance costs reflect short-term impacts until production is returned to pre-incident levels and includes the value of lost production and staff turnover costs. Value of lost production is measured by combining average duration of absence (by severity category) with average weekly earnings (AWEs), where AWE is a weighted income of two groups of employees permanent or fixed term and casual [30]. Cost of overtime reflects the proportion of overtime related to work-related injuries and wage of workers that would not be required if there were no injury. Overtime is valued by combining AWE with duration of absence (by severity category) and an average taxation rate of 40% [26]. The cost of replacing existing staff affected by work-related incidents is equivalent to 26 weeks of AWE, and the cost of training new staff in the event of full incapacity or a fatality is equivalent to 2.5 weeks of AWE [26].

#### 2.2.2. Human Capital Costs

This analysis uses the human capital approach to costing. Human capital costs consider the long-run costs, such as loss of potential output, occurring after a restoration of pre-incident production levels. They are calculated as a residual between total human capital loss and deadweight loss to society from taxation redistributions. For full incapacity or fatality, human capital costs are measured by considering the value of potential future earnings from time of injury to retirement age in Australia (i.e., 65 years) assuming a discount profile and productivity loss. The discount profile considers the likely changes in the value of money over time by including the opportunity cost of saving (4.58%) [31] and the rate of inflation (2.75%) [32]. A productivity factor of 1.75% [26] is applied to reflect long-term increases in AWE above the prevailing wage inflation rate. Tax losses due to foregone income are valued using a tax rate of 25% [33].

NCIS data is used to identify the average age of suicide. The median age of suicide is used as a proxy for the average age of a full incapacity case. For full incapacity, future earnings also include the average social welfare payments received, since these contribute to post-injury income. These costs are borne by the government through the disability support pension—equivalent to $777.50 per fortnight [34] (in 2014 dollars [32]). The average life expectancy at birth in Australia in 2014 is 82.4 years (84.4 years for females and 80.3 years for males) [35]. If the median age of suicide is 42.0 years (40.0 years for females and 42.0 years for males), then potential years of life lost (PYLL) are 40.4 years (44.4 for females and 38.3 for males). Potential productive years of life lost are 24.0 years (26.0 for females and 24.0 for males). It is assumed that an incapacitated person will receive the disability support pension from time of incident until average age of death. Further, it is assumed the full incapacitated and fatalities never return to work and the full cost is borne by the government in terms of lost income and tax revenue. These assumptions are consistent with those used in the Safe Work Australia report [26].

#### 2.2.3. Medical Costs

Medical costs are expenses incurred by workers and the community through medical treatment. Average medical costs per incident by severity are sourced from Safe Work Australia [26]: $820 per short absence; $12,515 per full incapacity case; and $2430 per fatality. In all work-related incidents involving medical care, the employer covers the first $500, and employers contribute 15% of the difference with the government accounting for the remainder.

#### 2.2.4. Administrative Costs

Administrative costs included in this analysis are investigation costs, travel costs, and funeral costs. Investigation costs consider the costs of investigating an incident and the administrative cost of collecting and reporting information on work-related incidents. Average investigation costs by severity are sourced from Safe Work Australia [26]: $28 per short absence; $2374 per full incapacity case; and $2840 per fatality. It is assumed that government investigation costs would be equal to the cost borne by the employer. Travel costs represent expenses for travel to doctors, rehabilitation centers, solicitors, etc.: $5 per short absence and $260 per full incapacity case. For full incapacity cases, the government is assumed to match travel expenses 1:1 with the individual, in effect assuming a 50% travel concession for full incapacitated workers. Funeral costs are estimated at $4000 and borne entirely by the worker (family). It is acknowledged that funeral costs may be associated with all deaths, fatality by suicide brings these costs forward.

#### 2.2.5. Other Costs

Other costs included in this analysis are cost of carers and aids/modifications for full incapacity cases and the cost of postvention services for fatalities. Postvention is a psychological first aid, crisis intervention, and other support offered after a suicide to affected individuals or the workplace as a whole to alleviate possible negative effects of the event. Safe Work Australia [26] uses disability support pension payments of $2056 and $646 per annum as a proxy for the cost of carers and the cost of aids and modifications, respectively. The total of these payments is discounted to present value terms over the period between the incident and reduced life expectancy.

A fatality by suicide has a flow-on effect with research suggesting that each fatality by suicide impacts directly on six to twenty people [36]. The economic cost associated with suicide bereavement has been estimated at $14,350 [36] (in 2014 dollars [32]). Evidence from an Industry source [23] suggests that each fatality by suicide may be witnessed by on average three colleagues that would then require counselling and time off work as part of postvention care. These costs are estimated at $10,000 per worker from time of incident to return to full duties [15]. This assumption is in line with other attempts to measure the ripple effects of a suicide but may be considered as conservative as it only considers the impact on workers and not families or friends.

#### 2.2.6. Transfer Costs

The redistribution of public sector resources to care for incapacitated persons incurs deadweight costs on society—for every dollar of tax raised, about 28.75 cents is absorbed in the distortions induced and the administration of the tax system. In this analysis the deadweight loss is measured as the value of taxation receipts foregone, equivalent to 28.75 cents in every foregone tax dollar [26].

Consistent with the Safe Work Australia report [26], the methodology used in this analysis is based on an incidence based approach. The incidence based approach allows a better estimate of the economic cost, since it allows the future costs for new cases to be followed over the expected lifetime of the case. This approach is known as the lifetime cost approach, and provides an indicator of the benefits of reducing work-related incidents. The costs that an injury imposes in future years are discounted to present values (i.e., constant 2014 dollars in this analysis). The lifetime cost approach assumes the levels and structures of current costs accurately reflect future costs.

A further assumption made in the Safe Work Australia report [26], and carried over to this analysis, is that the methodology is based on an ex-post approach in which costs are attributed to incidents after they occur and as a direct result of the incident. The nature of the compensation-based data, on which the Safe Work Australia report is based, lends itself to an ex-post estimation process. The current and future costs associated with each case can be assigned individually (since the number of cases and the nature of each case is known) and the total cost estimated by aggregating the cost of each case and/or cost component from the bottom-up.

### 2.3. Potential Impact of a Universal Workplace Suicide Prevention Intervention

Mates in Construction (MIC) is an example of a multifaceted workplace suicide prevention strategy developed in Australia [15,20,21]. MIC is a multimodal prevention and early intervention program, consistent with the national “living is for everyone” suicide prevention strategy and with Mrazek and Haggerty’s [37] spectrum of prevention and intervention. MIC has three main components: general awareness training (GAT); connector training; and applied suicide intervention skills training (ASIST) [24]. GAT involves a 1-h training session provided by accredited trainers to construction workers on sites with the aims of increasing awareness of suicide as a workplace health and safety issue, improving knowledge of warning signs, and encouraging workers to seek support. Connector training involves a 4-h training session provided by MIC. The role of a connector is to keep coworkers safe while connecting them to help, that is, to an ASIST-trained worker, MIC field officer, or case manager. ASIST workers undergo an intensive 2-day training course to enable them to identify cues and respond appropriately to calls for help with the goal of reaching a contract or safe plan involving extra help and safety. MIC accredited sites or employers also receive promotional materials and access to other MIC programs including a 24/7 helpline.

Gullestrup et al. (2011) [24] established the social validity and effectiveness of MIC for improving suicide and mental health awareness, help-seeking behavior, and treatment engagement. Doran et al. (2015) [20] extended the research by Gullestrup et al. (2011) [24] and assessed the impact of MIC by comparing the risk of suicide pre and post the implementation of MIC in the Queensland construction industry. This method of calculating a relative risk ratio (RRR) has also been used in an evaluation of the United States Air Force suicide prevention program [38]. MIC was introduced in Queensland in 2008—the pre-period refers to 2003–2007 and the post-period 2008–2012. Doran et al. (2015) [20] reported a RRR of 0.904 (95% confidence interval 0.909–0.900), equivalent to a reduction in suicide risk of 9.6% (9.1%–10.0%). However, this reduction in suicide risk was only partially attributed to MIC as there may have been other suicide prevention strategies that could have impacted on reduction in suicide risk. Over the post-MIC intervention period (2008–2012), the average uptake (or penetration) of general awareness training was 9.4% (i.e., the average proportion of the construction industry workforce exposed to GAT activities over the period of interest). Doran et al. (2015) considered that the introduction of MIC was associated with a 0.91% (9.6% × 9.4%) reduction in the risk of suicide.

This rate is used in the current analysis to estimate the potential impact of rolling out a similar workplace suicide prevention strategy, with a similar uptake as MIC. The cost of rolling out MIC in Queensland was estimated at $37.46 per worker each year. Using Australian Labor Market Statistics [39], the size of the employed Australian workforce is estimated at 11,582,797 in December 2014. Our analysis adopts an update rate of 9.4% to reflect the proportion of workers exposed to the workplace suicide prevention strategy. The potential economic impact of implementing MIC across the entire Australian workforce is derived by comparing the economic savings from fewer suicides (i.e., a 0.91% reduction in suicide) with the cost of implementing the program ($37.46 per worker per year). Results are expressed as a ratio of benefits to costs with the ratio greater than 1 representing a positive economic investment. Table 3 contains key parameters applied to the costing analysis and hypothetical impact modelling.

### 2.4. Sensitivity Analsysis

Sensitivity analyses were undertaken to test the robustness of results to changes in key parameters. The ratio of suicides to suicide attempts (i.e., 1:15) was adjusted to 1:10 and 1:20 reflecting the lower and upper boundaries of the WHO estimate [1]. The proportion of suicide attempts resulting in full incapacity (i.e., 17% of suicide attempts) was varied by 5 percentage points (±5%). Median age of suicide (i.e., 42 years) was reduced to 40 years depicting the female median age of suicide. Average life expectancy at birth (i.e., 82.4 years) was reduced to 71.6 years and 77.0 years reflecting the average life expectancy of people born 40 and 42 years ago. The discount rate used to convert future costs to present value (i.e., 4.58%) was adjusted to 0%, 3%, and 5%. Average weekly earnings (i.e., $1182) were reduced to $863 to accommodate the fact that around three-quarters of people who die by suicide would have had a mental disorder [40]. Evidence suggests that people with mental illnesses earn on average a third less than median earnings compared to those with no health condition in full time employment and adjusted for age, sex, and education [41]. Suicide risk reduction (i.e., 9.6%) was varied according to 95% confidence interval (9.1%–10.0%). The attribution of MIC to avert suicide was increased by 5 and 10 percentage points of 9.4%. Lastly, the average cost of MIC per worker per year (i.e., $37.46) was increased to $45.00 after discussion with the MIC board.

### 2.5. Ethics

Ethics approval to use NCIS data was granted by the Victorian Department of Justice (Project identification code: M0366) and the Central Queensland University Human Research Ethics Committees (H16/04-085).

## 3. Results

### 3.1. Suicide and Non-Fatal Suicide Attempts

In 2014, a total of 2419 Australians died from suicide, suggesting an age adjusted death rate of 10.3 per 100,000. Of these fatalities by suicide, 903 were employed at the time of death. Using the WHO statement [1] on the relationship between suicide and NFSB, we could expect 13,545 non-fatal suicide attempts with 2303 resulting in full incapacity and 11,242 resulting in a short absence from work.

Table 4 provides various characteristics of these 903 people. Eighty four percent (*n* = 759) were males; the median age at time of death was 41.1 years for females and 42.1 years for males; 43% were married/de facto; threats to breathing were the most common cause of death (66%); and the majority of deaths (62%) occurred at home. There was no significant differences in age, marital status, or place of death. There were, however, significant differences in the cause of death between females and males $\left(\chi^{2}\left(6,\;n=903\right)=34.332,p<0.001\right)$. Threats to breathing were the most common cause of death in males (M:F, 68% vs. 57%), whereas poisoning was more common in females (F:M, 32% vs. 13%).

### 3.2. Average Cost of Suicide and Non-Fatal Suicide Attempts

The average cost associated with suicide incidents and NFSB is provided in Table 5. The average cost of a short-term absence is estimated at $1184 per incident; $2.25 million per incident resulting in full incapacity; and $1.69 million for each fatality. The key cost driver in both full incapacity cases and a fatality is lost income (and taxes) and, for full incapacity only, the additional cost of welfare payments.

### 3.3. Total Cost of Suicide and Non-Fatal Suicide Attempts

Total cost associated with NFSB and suicide is provided in Table 6. The total cost of suicide and NFSB in 2014 is estimated at $6.73 billion. The majority of this cost is attributed to the cost associated with NFSB resulting in full incapacity (77.3% of total costs or $5.19 billion), followed by the cost of a suicide (22.5% of total costs or $1.52 billion) and NFSB resulting in a short absence from work (0.2% of total costs or $13.31 million).

### 3.4. Potential Impact of a Universal Workplace Suicide Prevention Intervention

The potential economic impact of implementing the multifaceted workplace suicide prevention strategy (MIC) across the Australian workforce has an estimated saving of $61.26 million each year (Table 7). The majority of benefits (97%) are estimated to flow to the government with a saving of $59.44 million each year. The total annual cost of implementing the program is estimated at $40.97 million, suggesting a benefit cost ratio equivalent to 1.50:1, representing a positive economic investment of public funds.

### 3.5. Sensitivity Analysis

Table 8 provides the results of sensitivity analyses. All variations in key parameters have little impact on the positive economic benefit of MIC, resulting in the benefit-cost ratio ranging between 1.11 and 3.07.

## 4. Discussion

The objective of this study has been to quantify the economic cost of suicide and NFSB among employed Australians and to examine the potential impact of introducing a workplace suicide prevention intervention to reduce this burden. Although evidence suggests that the rate of suicide among workers is high, to the best of our knowledge, this is the first study that has quantified the cost and potential economic return of addressing this harm through a workplace strategy. However, before discussing the key findings of this research, it is important to reflect on the strengths and limitations of our approach.

This study has several notable strengths. First, our costing methodology is consistent with the Safe Work Australia approach [26,27] which had been endorsed by the National Occupational Health and Safety Commission [23] and applied in several studies [18,21]. Such a framework adds validity and robustness to the cost estimates. Second, this analysis uses the best available evidence of suicide fatalities in Australia, recorded in the NCIS. The system underlying cause-of-death statistics is complex and suicide is a particularly challenging cause to record and classify [42]. The NCIS provides confidence that our data is accurate [43,44]. Third, we have used evidence of an existing workplace intervention to model the potential economic impact of implementing a universal workplace strategy. We have erred on the side of caution by adopting conservative estimates of the potential effectiveness and reach of such a workplace strategy. Fourth, unlike most suicide costing studies, our analysis includes postvention costs associated with bereavement and counselling. Failure to include postvention services is known to underestimate any cost estimates [45].

Several limitations are also worth noting. First, given a lack of good quality Australian data on NFSB, we have used the World Health Organization ratio of 15 cases of NFSB to every death by suicide to approximate the number of non-fatal attempts. Although this relationship is supported by Australian research [28,29], there may be scope to either under or overestimate cases of NFSB. Second, the costing analysis relies on averages—average weekly earnings and median age of death. These averages will mask the potential variance in economic cost per fatality with younger people having a higher economic cost than the elderly. Third, the NCIS does not contain information on full-time or part-time status, though the average weekly earnings figures took into account a weighted income for permanent or fixed term and casual employees. Fourth, the effectiveness parameters are based on a pre-post study without control group [21]. Fifth, the analysis did not attempt to estimate the costs saved by the transfer of knowledge gained through workplace training such as MIC. The ripple effects of other suicide gatekeeper programs have shown that for each person trained another five people have conversations with that trainee and learn about best practices in suicide intervention [28,45]. This transfer of knowledge then increases the potential of saving lives outside of work. Lastly, the cost calculations do not include potential savings of suicide, as advocated by Yang and Lester [6], which might arise as a result of not having to treat the depressive and other psychiatric disorders of those who kill themselves; or avoidance of pension, social security, and nursing home care costs.

Our key findings suggest that, in 2014, over one-third of all suicide fatalities in Australia were among employed people. An estimated 13,545 workers experienced NFSB with 2303 resulting in full incapacity and 11,242 resulting in a short absence from work. The present value of the economic cost of suicide and NFSB is over $6.73 billion. Across all categories, the burden of cost associated with self-harm and suicide is borne largely by the government: 97% or $6.56 billion of the total combined cost of $6.73 billion.

Death by suicide for employed people is known to be a result of a complex interaction between individual vulnerabilities and work-related environmental factors that trigger stress reactions and contribute to poor mental wellbeing [1]. In its recent call for action, Suicide Prevention Australia argued the urgent need to addressing a range of systemic issues in the workplace, including managing unemployment, workers’ compensation, and coronial processes [28]. Our analysis suggests that if a workplace strategy such as MIC was universally implemented, there might be on average 8.2 fewer suicides, 21.0 fewer self-harm attempts ending in full incapacity, and 102.3 fewer self-harm attempts ending in a short absence from work. The potential economic benefit of averting this harm ($61.26 million each year) considerably outweighs the cost of the strategy ($40.97 million each year). For every one dollar invested in a workplace program like MIC, the benefits would be in excess of $1.50 ($1.11–$3.07), representing a positive economic investment. All variations of the key parameter in sensitivity analyses hold the positive benefit-cost ratio. These results support the view that the level of return on investment (i.e., $1.50) is a conservative estimate. Our estimates of the cost of suicide and NFSB in the Australian workforce reinforce the importance of additional preventive measures.

## 5. Conclusions

Rates of suicide and NFSB are far too high in Australia and elsewhere. Although being employed has a protective effect on suicide behavior, over one-third of all Australian suicide fatalities during 2014 were among employed people. The associated economic burden of $6.73 billion is avoidable. More needs to be done to reduce this burden. Although workplace strategies are appropriate for those employed, these interventions must be used within a multifaceted approach that reflects the complex nature of self-harming behavior.

## Figures and Tables

**Table 1:** Safe Work Australia categories of severity.

Category Label	Severity	Category Definition
Short absence	Less than five days off work	A minor work-related injury or illness, involving less than five working days absence from normal duties, where the worker was able to return to full duties
Long absence	Five days or more off work and return to work on full duties	A minor work-related injury or illness, involving five or more working days and less than six months off work, where the worker was able to return to full duties
Partial incapacity	Five days or more off work and return to work on reduced duties or lower income	A work-related injury or illness which results in the worker returning to work more than six months after first leaving work
Full incapacity	Permanently incapacitated with no return to work	A work-related injury or disease, which results in the individual being permanently unable to return to work
Fatality	Fatality	A work-related injury or disease, which results in death

Source: Safe Work Australia, 2015 [26].

**Table 2:** Economic cost borne by the employer, worker, and government.

Conceptual Group	Cost Item	Employer	Worker	Government
Production disturbance costs	Value of lost production	Overtime premium and value of wages paid while away from work	Zero	Zero
	Staff turnover costs	Staff turnover costs	Zero	Zero
Human capital costs	Net present value of lost earnings	Zero	Zero	Loss of income and welfare payments transferred to worker for loss of wage minus deadweight loss associated with tax revenue forgone
Medical costs	Medical and rehabilitation costs	Threshold medical payments	Gap payments	Medical payments not covered by employer or worker
Admin. costs	Investigation costs	Employer investigation costs	Zero	Costs of running the compensation system (including investigation claims)
	Travel costs	Zero	Out of pocket expenses	Compensation for travel costs
	Funeral costs	Zero	Out of pocket expenses	Zero
Other	Carers	Zero	Zero	Payments to carers
	Aids, equipment, and modifications	Zero	Zero	Reimbursements for aids, equipment, and modifications
	Postvention	Postvention	Zero	Postvention
Transfer costs	Deadweight costs of tax revenue foregone	Zero	Zero	Deadweight costs of tax revenue foregone

Source: Safe Work Australia, 2015 [26].

**Table 3:** Summary of key assumptions.

Item	Description	Estimate		
		Female	Male	Person
**Suicide fatalities**	National Coronial Information System (NCIS ) from 1 January 2014–31 December 2014 and classified as intentional self-harm [25]	144	759	903
**Non-fatal suicide behavior**				
Non-fatal suicide attempts	World Health Organization estimates 15 suicide attempts for one fatality [1]	2160	11,385	13,545
Attempts resulting in full incapacity	Safe Work Australia estimates 17% of all suicide attempts result in full incapacity [26]	367	1935	2303
Attempts resulting in short absence	Safe Work Australia estimates 83% of all suicide attempts result in short absence from work [26]	1793	9450	11,242
**Suicide indicator data**				
Median age of suicide	NCIS from 1 January 2014–31 December 2014 and classified as intentional self-harm [25]	40.0	42.0	42.0
Average life expectancy at birth	Australian Bureau of Statistics (ABS) Deaths, Australia, 2014 [4]	84.4	80.3	82.4
Potential years of life lost (YLL)	(Average life expectancy at birth [4]–Median age of suicide [25])	44.4	38.3	40.4
Potential productive years of life lost (PYLL)	(Average retirement age in Australia–Median age of suicide [25])	26.0	24.0	24.0
**Costing analysis**				
Average earnings	Proxy for productivity, ABS 6306.0 Employee Earnings and Hours survey, May 2014: Average weekly earnings as a weighted earnings for permanent or fixed term and casual employees by gender [30]	$940	$1430	$1182
Discount rate	Opportunity cost of money: Average of rates of return for private and government saving instruments and Reserve Bank of Australia (RBA) target for March 2005 to December 2014 [31]			4.58%
Inflation rate	Average of annual weighted ABS 6401.0 Consumer Price Index (CPI) from December 2004 to December 2014 [32]			2.75%
Productivity rate	Annual increase in workers’ productivity. Safe Work Australia report 2012–2013 [26]			1.75%
Average tax for foregone earnings	Australian Taxation Office [33]			25.00%
Transfer costs	Deadweight cost of welfare payments and tax losses. Safe Work Australia report 2012–2013 [26]			28.75%
Welfare for disability per year	Disability support pension $797.90 per fortnight [34] adjusted to 2014 dollars using CPI correction factor [32]			$20,214
Carers—full incapacity cases only from incident to end of life	Estimated applicable Disability Support Pension payments of $2056 per annum, discounted to present value over the period between the incident and reduced life expectancy. Safe Work Australia report 2012–2013 [26]			$2056
Aids and modifications—full incapacity cases only from incident to end of life	Estimated applicable Disability Support Pension payments of $646 per annum, discounted to present value over the period between the incident and reduced life expectancy. Safe Work Australia report 2012–2013 [26]			$646
**Impact analysis**				
Average cost MIC per worker per year	Mates in Construction (MIC) estimates [21]			$37.46
Average size of employed population	ABS Labor force: Employed total, persons December 2014 [39]			11,582,797
Average penetration of MIC	Mates in Construction estimates [21]			9.40%
Average workers exposed each year	(Average size of employed population [39] × Average penetration of MIC [21])			1,093,791
Total cost MIC per worker per year	(Average workers exposed each year × Average cost MIC per worker per year [21])			$40,973,427

**Table 4:** Profile of fatality by suicide for those employed.

Variable	Female (*n* = 144) %	Male (*n* = 759) %	Total (*n* = 903) No (%)	*p*-Value ($\chi^{2}$ or *t* Test)
Mean age in years (SD)	41.1 (12.4)	42.1 (12.5)	41.9 (12.5)	0.392
Median age in years (SD)	40.0 (12.4)	42.0 (12.5)	42.0 (12.5)	
Marital status				0.871
Married/de facto	44	43	388 (43%)	
Divorced	9	7	66 (7%)	
Separated	13	16	144 (16%)	
Widowed	1	1	5 (1%)	
Never married	23	24	218 (24%)	
Missing data	10	9	82 (9%)	
Cause of death				<0.001
Threats to breathing	57	68	596 (66%)	
Poisoning	32	13	147 (16%)	
Blunt force	6	8	66 (7%)	
Piercing, penetrating force	4	9	78 (9%)	
Thermal mechanism	1	1	5 (1%)	
Other	0	1	10 (1%)	
Missing data	0	0	1 (0%)	
Suicide location				0.270
Home	67	62	564 (62%)	
Commercial area (non-recreational incl. construction area)	3	6	50 (6%)	
Farm or countryside	7	10	86 (10%)	
Public area (incl. transport)	11	13	113 (12%)	
Medical/residential facilities and other institutions	12	7	73 (8%)	
School, educational, or sports area	0	1	5 (1%)	
Other place of occurrence (mainly bridge)	0	1	11 (1%)	
Missing data	0	0	1 (0%)	

*n* = 1 case with unknown gender.

**Table 5:** Average cost of suicide and non-fatal suicide behavior (NFSB) by gender, 2014.

Cost Category	Female	Male	Person
NFSB resulting in short absence			
Production disturbance costs	$263	$400	$331
Human capital costs	$0	$0	$0
Medical costs	$820	$820	$820
Administrative costs	$33	$33	$33
Other	$0	$0	$0
Transfer costs	$0	$0	$0
Subtotal	$1116	$1253	$1184
NFSB resulting in full incapacity			
Production disturbance costs	$37,120	$38,902	$38,001
Human capital costs	$1,849,220	$2,289,329	$2,013,011
Medical costs	$12,515	$12,515	$12,515
Administrative costs	$2634	$2634	$2634
Other	$85,071	$77,565	$80,158
Transfer costs	$93,919	$132,350	$109,449
Subtotal	$2,080,479	$2,553,295	$2,255,769
Suicide			
Production disturbance costs	$37,120	$38,902	$38,001
Human capital costs	$1,212,782	$1,709,042	$1,413,325
Medical costs	$2430	$2430	$2430
Administrative costs	$7030	$7030	$7030
Other	$116,100	$116,100	$116,100
Transfer costs	$93,919	$132,350	$109,449
Subtotal	$1,469,381	$2,005,854	$1,686,335

**Table 6:** Total cost of suicide and NFSB by gender, 2014.

Cost Category	Females	Males	Persons
NFSB resulting in short absence			
Production disturbance costs	$472,018	$3,783,251	$3,721,911
Human capital costs	$0	$0	$0
Medical costs	$1,470,260	$7,749,000	$9,218,440
Administrative costs	$59,169	$311,850	$370,986
Other	$0	$0	$0
Transfer costs	$0	$0	$0
Subtotal	$2,001,447	$11,844,101	$13,311,337
NFSB resulting in full incapacity			
Production disturbance costs	$13,622,940	$75,275,122	$87,517,077
Human capital costs	$678,663,734	$4,429,851,230	$4,635,964,426
Medical costs	$4,593,005	$24,216,525	$28,822,045
Administrative costs	$966,678	$5,096,790	$6,066,102
Other	$31,220,935	$150,088,431	$184,604,324
Transfer costs	$34,468,322	$256,097,373	$252,061,909
Subtotal	$763,535,614	$4,940,625,472	$5,195,035,883
Suicide			
Production disturbance costs	$5,345,241	$29,526,521	$34,315,206
Human capital costs	$174,640,587	$1,297,162,972	$1,276,232,047
Medical costs	$349,920	$1,844,370	$2,194,290
Administrative costs	$1,012,320	$5,335,770	$6,348,090
Other	$16,718,400	$88,119,900	$104,838,300
Transfer costs	$13,524,355	$100,453,698	$98,832,785
Subtotal	$211,590,823	$1,522,443,231	$1,522,760,718
Total	$977,127,884	$6,474,912,804	$6,731,107,939

**Table 7:** Potential economic savings of a workplace suicide prevention strategy in one year.

Type of Incident	Number of Incidents Reduced by MIC Each Year	Average Cost Per Incident	Total Cost Savings	% Savings to Government
Short absence	102.3	$1184	$121,163	24%
Full incapacity	21.0	$2,255,769	$47,277,964	98%
Fatality	8.2	$1,686,335	$13,860,149	96%
Total	131.5		$61,259,276	97%

**Table 8:** Sensitivity analysis of key parameters (in Australian dollars).

Parameter Varied	Number of Averted Incidents	Economic Savings Per Year	Cost of the Suicide Prevention Strategy (MIC)	Benefit-Cost Ratio
Ratio of suicides to suicide attempts				
Sensitivity 1 = 1:10	90.4	$45,459,567	$40,973,427	1.11
Baseline = 1:15	131.5	$61,259,276	$40,973,427	1.50
Sensitivity 2 = 1:20	172.6	$77,058,986	$40,973,427	1.88
Proportion of suicide attempts resulting in full incapacity				
Sensitivity 3 = 12%	131.5	$47,361,292	$40,973,427	1.16
Baseline = 17%	131.5	$61,259,276	$40,973,427	1.50
Sensitivity 4 = 22%	131.5	$75,157,261	$40,973,427	1.83
Median age of suicide				
Sensitivity 5 = 40	131.5	$65,220,880	$40,973,427	1.59
Baseline = 42	131.5	$61,259,276	$40,973,427	1.50
Average life expectancy at birth				
Sensitivity 6 = 71.6	131.5	$58,504,155	$40,973,427	1.43
Sensitivity 7 = 77.0	131.5	$59,824,752	$40,973,427	1.46
Baseline = 82.4	131.5	$61,259,276	$40,973,427	1.50
Discount rate used to convert future costs to present value				
Sensitivity 8 = 0%	131.5	$121,813,872	$40,973,427	2.97
Sensitivity 9 = 3%	131.5	$75,700,819	$40,973,427	1.85
Baseline = 4.58%	131.5	$61,259,276	$40,973,427	1.50
Sensitivity 10 = 5%	131.5	$58,173,390	$40,973,427	1.42
Average earnings				
Sensitivity 11 = $863	131.5	$49,219,807	$40,973,427	1.20
Baseline = $1182	131.5	$61,259,276	$40,973,427	1.50
Suicide risk reduction				
Sensitivity 12 = 9.1%	124.2	$57,835,982	$40,973,427	1.41
Baseline = 9.6%	131.5	$61,259,276	$40,973,427	1.50
Sensitivity 13 = 10.0%	136.4	$63,556,024	$40,973,427	1.55
Attribution of MIC to avert suicide				
Baseline = 9.4%	131.5	$61,259,276	$40,973,427	1.50
Sensitivity 14 = 14.4%	200.5	$93,414,281	$40,973,427	2.28
Sensitivity 15 = 19.4%	270.2	$125,849,795	$40,973,427	3.07
Average cost of MIC per worker per year				
Baseline = $37.46	131.5	$61,259,276	$40,973,427	1.50
Sensitivity 16 = $45.00	131.5	$61,259,276	$49,220,615	1.24
